# A novel method for evaluating mastoid defect regrowth after cochlear implantation

**DOI:** 10.1038/s41598-024-59295-x

**Published:** 2024-04-22

**Authors:** Nezar Hamed, Asma Alahmadi, Yassin Abdelsamad, Abdulaziz Alballaa, Fida Almuhawas, Hussain Allami, Hisham Almousa, Abdulrahman Hagr

**Affiliations:** 1 King Abdullah Ear Specialist Center (KAESC), Department of Otorhinolaryngology, College of Medicine, King Saud University Medical City (KSUMC), King Saud University, P.O. Box: 245, 11411 Riyadh, Saudi Arabia; 2 Research Department, MED-EL GmbH, Riyadh, Saudi Arabia

**Keywords:** Outcomes research, Paediatric research, Medical imaging

## Abstract

This retrospective study examined mastoid defects resulting from cochlear implant (CI) surgery and their potential for spontaneous regrowth across different age groups. Spontaneous closure of mastoid defects has been observed in certain CI patients during revision surgery or through post-operative temporal bone computer tomography (TB-CT). The analysis encompassed 123 CI recipients, comprising 81.3% children and 18.7% adults, who underwent post-operative TB-CT scans. Using image adjustment software, the study measured mastoid defect areas and found a significant reduction in children's defects between the initial and subsequent scans. Notably, mastoid defect areas differed significantly between children and adults at both time points. Furthermore, the analysis revealed significant correlations between mastoid defect areas and the age at implantation as well as the time elapsed since the CI surgery and the first CT scan. This study provides valuable insights for evaluating CI patients scheduled for revision surgery by assessing potential surgical challenges and duration. Furthermore, it may have a pivotal role in evaluating patients who experience postauricular swelling subsequent to CI surgery.

## Introduction

Cochlear implantation is an innovative and highly effective intervention that not only improves the auditory capabilities for individuals with severe to profound hearing loss but also enhances their overall quality of life. While the focus of cochlear implant (CI) research has primarily been on hearing and speech outcomes, another important aspect that warrants consideration is the assessment of mastoid defects resulting from the surgical procedure and the potential for mastoid regrowth. This defect has been noticed in some CI patients that it had spontaneously closed during the revision surgery or radiologically in post-operative temporal bone computer tomography (TB-CT).

Mastoid development and pneumatization exhibit comparable growth patterns across different ethnic groups. At one year of age, the mastoid area measures approximately 3.5–4 cm^2^, with a relatively rapid growth rate. Subsequently, the mastoid area undergoes around 50% reduction in the growth rate until the age of 6–7. This is followed by a slower growth rate during puberty, ultimately reaching the adult size of about 12 cm^2^^1^. Histologically, the mastoid bone comprised of two distinct layers. The outer layer is composed of cortical (compact) bone, which provides strength and protection^2^. In contrast, the inner layer consists of cancellous (spongy) bone, characterized by its trabecular structure and interconnected spaces. This combination of compact and cancellous bone in the mastoid process facilitates its diverse functions, including providing support to surrounding structures and contributing to sound transmission and resonance. In terms of healing, compact bone demonstrates a slower healing capacity relative to cancellous bone, primarily attributable to diminished blood supply and decreased cellular activity. Generally, the healing time for bone fractures can vary depending on various factors^3–5^.

Mastoid defects refer to the bony openings that remain in the mastoid process following cochlear implantation. These defects occur as a result of the retro-auricular canal wall-up mastoidectomy procedure, which is performed to access the round window (RW) through posterior tympanotomy, as described by House^6,7^. On the other hand, a recent study has revealed that Intact-canal-wall mastoidectomy operations can potentially result in a cosmetically undesirable depression in the postauricular region, especially when external sound processors are used behind the ear^8^. However, the use of autogenous mastoid cortical bone cap for covering mastoidectomy defects during CI surgery demonstrate successful reconstruction of the mastoidectomy defects, without any postauricular depressions or complications such as wound infection or intracranial issues postoperatively^9^. This draws attention to the potential promising outcomes of natural mastoid defect closure following cochlear implantation.

The significance of revision CI surgery is progressively growing within the Otology field. With the ongoing global increase in the number of CI surgeries, there is a corresponding rise in the incidence of revision procedures, whether caused by device failures or complications that are not directly related to the device itself. Moreover, it is worth noting that reimplantation rates tend to be higher in children than in adults^10–13^. During revision CI surgery, several surgical challenges may be encountered, such as the closure of the mastoid defect, neo-osteogenesis and fibrosis, which can result in device fixation^14^. These difficulties can lead to prolonged surgery duration and increased exposure to anesthesia, as drilling may be required to reopen the mastoid cavity^15^. Furthermore, Preserving the integrity of the CI device is of utmost importance as it allows for complete removal and minimizes the risk of complications. Notably, maintaining device integrity enables comprehensive investigation by the manufacturer.

The risk of infection is highest shortly after CI surgery, but it can persist for up to two years following the procedure^16^. Nevertheless, infections in CI patients are rare, necessitating prompt medical and surgical intervention to prevent complications. Complications exhibited a 3.2% incidence in children and 2.0% in adults, with the highest rates observed among children aged between 1 and 2 years. Pediatric complications often arose within 6 months post-CI surgery, displaying a recurrence rate of 22% compared to 14% in adults. Prosthetic inflammation/infection emerged as the most common complication, followed by mastoiditis and cellulitis, while instances of meningitis were less frequent^17^. In addition, These infections can potentially lead to a decline in audiological performance and, in severe cases, may necessitate explantation and reimplantation procedures^18^. Furthermore, Single-layer or double-layer soft tissue flaps play a crucial role in ensuring the success of CI surgery. The decision to utilize either a single-layer or double-layer soft tissue flap in CI procedures is contingent upon considerations such as reach, pliability, and efficacy in managing exposed or infected implants. Success rates, which may reach up to 52.6% with a double-layer flap, are pivotal factors influencing this decision-making process^19^. Postauricular swelling commonly occurs due to acute otitis media^20^, Which may be related to bony defect in the cortical mastoid, allowing infection to spread towards the skin. However, it is essential to consider non-surgical mastoiditis as a potential cause of post-auricular swelling when natural mastoid defect regrowth and closure take place after a significant period of time post-implantation.

The exploration of mastoid defects in the existing literature has been inadequately addressed, with limited attention and dedicated research devoted to this matter. Therefore, the aim of this study was to fill this gap by conducting a comprehensive radiological evaluation of mastoid defect regrowth following cochlear implantation, considering diverse age groups.

## Materials and methods

### Study design

In this retrospective study, we carefully extracted data from medical records at our specialized referral center for neuro-otology and CI surgery. The research adhered rigorously to the ethical principles set out in the Declaration of Helsinki and obtained formal approval from the Institutional Review Board at King Saud University, denoted by Reference Number 22/0911/IRB. It's worth noting that the requirement for informed consent was formally waived due to the retrospective nature of the study.

### Patient selection

The study encompassed all patients who underwent CI surgery at our center during the period from July 2002 to November 2021. The inclusion criteria involved individuals of diverse ages and genders who regularly attended follow-up appointments at our clinic for at least one year after their cochlear implantation. Typically, the follow-up protocol for patients with CIs involves regular postoperative visits for monitoring and adjustment of the device settings, as well as assessing the overall performance and condition of the CI. This may include scheduled appointments at specific intervals, such as 1 week, 1 month, 3 months, 6 months, and annually following surgery. Additionally, audiologic assessments, such as speech perception tests and evaluation of device functionality, are conducted during these visits. It's important to note that the follow-up regimen may be altered if new symptoms or complications arise, warranting closer monitoring and management through more frequent appointments. Furthermore, the study required the availability of one or more post-operative high-resolution TB-CT scans for each participant. Patients with temporal bone fractures, pre-existing temporal bone diseases, or those who had undergone revision surgeries for any reason were excluded from the study. Our center followed a standard surgical approach for cochlear implantation, which involved a single-stage technique comprising cortical mastoidectomy and posterior tympanotomy.

### Radiological measurement of mastoid defect

**Figure 1:**
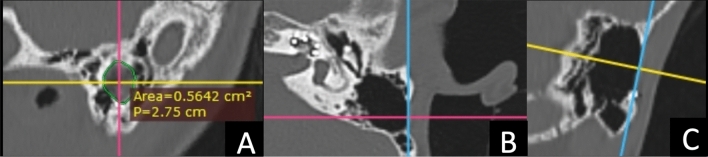
An illustration depicting the technique for measuring mastoid defects in temporal bone CT scans at the oblique sagittal plane (mastoid defect view). (**A**) Mastoid defect surface area measurement at its outer border in centimeter square. (**B**) The technique of adjusting the blue line on the anterior and posterior edges of the mastoid defect at the axial plane. (**C**) The technique of adjusting the pink line on the superior and inferior edges of the mastoid defect at the coronal plane.

All post-operative TB-CT scans has been collected from our database. The scans were obtained using a 512-slice multidetector-row CT scanner (General Electric Healthcare, Milwaukee, WI) with a slice thickness of 0.625 mm, 230 mAs, 140 kV, and a rotation time of 1 s. The images were reconstructed in the axial, coronal and sagittal views with a 0.3 mm interval. We utilized the RadiAnt DICOM Viewer 2022.1.1 software for image adjustment. The software allowed us to adjust the TB-CT images in different views, including the originally reconstructed planes. We particularly focused on optimizing the mastoid defect view in the oblique sagittal plane, which provided the best visualization of the mastoid defect. The mastoid defect view is defined as employing the sagittal oblique reformat technique, wherein the axial plane image is reformatted to reveal the longest line connecting the midpoint of the posterior wall of the external auditory canal to the midpoint of the posterior edge of the mastoid defect, approximately 10° from the coronal plane. Similarly, in the coronal plane, the image is reformatted to display the longest line connecting the midpoint of the superior and inferior edges of the mastoid defect, approximately 15° from the axial plane. We measured the mastoid defect at its most lateral bony border using the software (Fig. 1). The area of the defect was measured in square centimeters (cm^2^) using a closed polygonal measuring tool. It was not feasible to standardize the oblique sagittal view degree in all patients due to the variations in mastoid growth across different age groups. Furthermore, postoperative TB-CT imaging was employed to evaluate the placement of the electrode array in each cochlea.

The study population was categorized into two distinct groups: adults (> 18 years) and children (≤ 18 years). Unfortunately, there is a scarcity of literature documenting the average surface area of the mastoid defect following mastoidectomy. To bridge this knowledge gap, we divided our patients into subgroups based on the duration between cochlear implantation and the initial TB-CT scan, employing the average duration of bone fracture healing as a reference^4,5^. Subsequently, we measured the mastoid defect area in all patients who underwent TB-CT within 8 weeks post-cochlear implantation, enabling us to estimate the average mastoid defect area after CI surgery in both the adult and children. For patients who had the TB-CT scan beyond the 8-week timeframe, the mastoid defect area was also measured, and the mean difference between the subgroups was calculated. The CI patients were divided into three subgroups based on the surface area of the mastoid defect: large, denoting an area larger than 2 cm^2^; medium, ranging from 1 to 2 cm^2^; and small, indicating an area smaller than 1 cm^2^.

### Statistical analysis

Data were coded and analyzed using the Statistical Package for Social Science (IBM SPSS Statistics for mac, version 23 (IBM Corp., Armonk, N.Y., USA)). Data normality was assessed using the Kolmogorov–Smirnov and Shapiro–Wilk tests. Categorical data such as laterality and ear side of the implant were presented as frequencies and percentages. Continuous variables such as age at implantation and duration between post-CI and 1st CT were presented by the mean and standard deviation (SD). The association between categorical variables was tested using Chi-square and Fisher-exact tests, while the difference between 1st and 2nd CT in terms of mastoid defect area was evaluated using Wilcoxon signed-rank test. The difference between children and adults in terms of mastoid area defect was evaluated using the Mann–Whitney test. A p-value of less than 0.05 was considered significant.

### Ethics declarations

The study was rigorously carried out in complete accordance with the ethical guidelines delineated in the Declaration of Helsinki. Additionally, it received formal approval from the Institutional Review Board at King Saud University, denoted by Approval Number 22/0911/IRB.

### Consent for publication

Informed consent was officially waived by the same authoritative local committee, a decision made in recognition of the study's retrospective nature.

## Results

The study involved 123 patients, with a total of 181 implanted ears. Among these, 100 (81.3%) were children, and the remaining 23 (18.7%) were adults. The mean age at the time of implantation was 4.1 ± 3.0 years for children and 34.5 ± 11.9 years for adults. Of the right ears that were implanted, approximately 81.9% were in children, and 18.1% were in adults. The first CT scan was performed less than eight weeks after implantation for 25.41% of individuals, while 74.59% had the scan more than eight weeks after implantation. Out of the total sample, only 22 individuals underwent a second CT scan, with an average duration of 151.08 ± 128.66 weeks between the first and second scans. Furthermore, 90.9% of these individuals had the second CT scan more than eight weeks after the initial scan, as shown in Table 1.

**Table 1:** Demographic and implant characteristics.

		Children (≤ 18 Years) $N=100$ (152 ears)	Adult (> 18 Years) $N=23$ (29 ears)	Total $N=123$ (181 ears)
Side	Left	75 (86.2%)	12 (13.8%)	87 (48.07%)
	Right	77 (81.9%)	17 (18.1%)	94 (51.93%)
Age at implantation (years)		4.1 ± 3.0	34.5 ± 11.9	8.44 ± 12.44
Age at 1st CT (years)		6.3 ± 3.9	36.9 ± 11.0	11.2 ± 12.6
Duration between cochlear implantation and 1st CT (months)		24.4 ± 25.5	25.8 ± 43.2	27.2 ± 31.2
Number of implanted ears with 1st CT scan ≤ 8 weeks post CI surgery		37 (24.3%)	9 (31.03%)	46 (25.41%)
Number of implanted ears with 1st CT scan > 8 weeks post CI surgery		115 (75.7%)	20 (68.96%)	135 (74.59%)
Number of implanted ears with 2nd CT post CI surgery		18	4	22
Duration between 1st CT and 2nd CT (months)		41.0 ± 28.8	5.1 ± 5.1	34.9 ± 29.7

### Mastoid defect area

The mastoid defect area at the first CT scan was 1.336 ± 0.834 cm^2^ for children and 2.168 ± 0.376 cm^2^ for adults, with a mean area of 1.487 ± 0.831 cm^2^ for the total sample. At the second CT, the mastoid defect area was 0.753 ± 0.477 cm^2^ for children, 2.015 ± 0.388 cm^2^ for adults, and 0.983 ± 0.674 cm^2^ for the total sample. In children, the difference between the first and second CT in terms of mastoid defect area was statistically significant (MD = − 0.582, 95% CI − 0.95 to − 0.22; p < 0.001), while in the adults, the difference was not significant (MD = − 0.152, 95% CI − 0.38 to 0.081; p = 0.068), as shown in Table 2. The difference between children and adults in terms of mastoid area defect at 1st and 2nd CT was statistically significant (p < 0.001 and p = 0.002), respectively. However, the mean difference between the 1st and 2nd CT was comparable in both groups (p < 0.001).

**Table 2:** The mean mastoid defect area at the 1st and 2nd CT (cm^2^).

	1st CT		2nd CT		Mean difference	95% CI	p-value*
	Mean	SD	Mean	SD			
Child (N = 18)	1.336	0.834	0.753	0.477	− 0.582	− 0.95 to − 0.22	**< 0.001**
Adult (N = 4)	2.168	0.376	2.015	0.388	− 0.152	− 0.38 to 0.081	0.068
Total (N = 22)	1.487	0.831	0.983	0.674	− 0.504	− 0.81 to − 0.20	**< 0.001**
p-value^&^	**< 0.001**		**0.002**		0.166		

*p-value of the difference between 1st and 2nd CT; calculated by Wilcoxon-Signed test.

^&^p-value of the difference between children and adults; calculated by Mann–Whitney test.

Bold values indicate statistically significant p-values.

**Figure 2:**
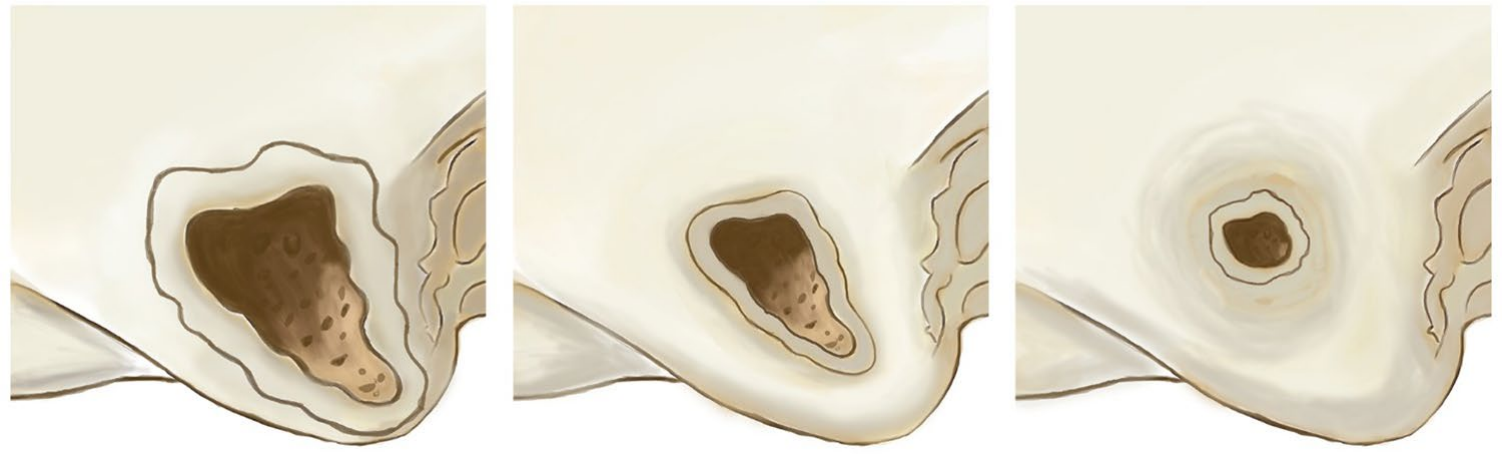
Description of mastoid defect regrowth after mastoidectomy overtime. Ordered according to the surface area of the mastoid defect into large, medium, and small respectively. large, denoting an area larger than 2 cm^2^; medium, ranging from 1 to 2 cm^2^; and small, indicating an area smaller than 1 cm^2^.

**Figure 3:**
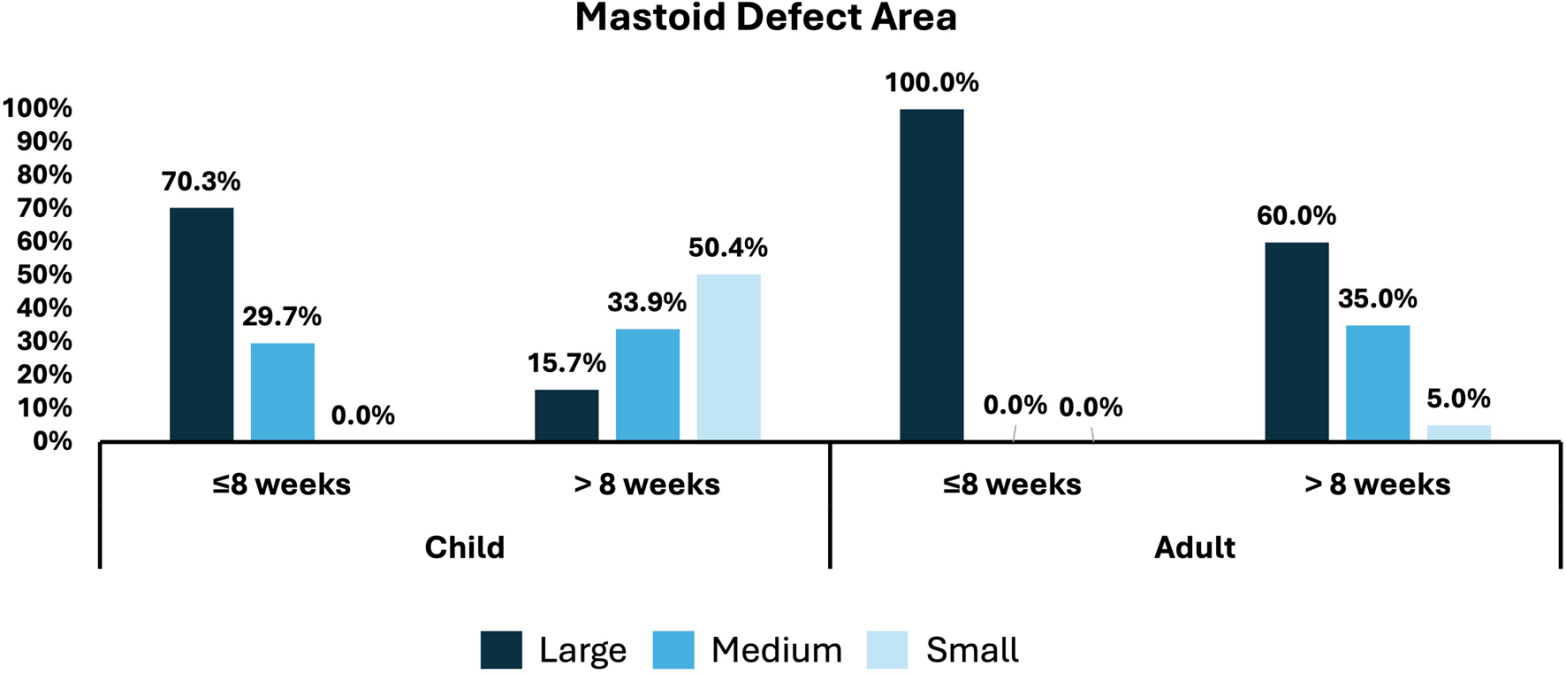
Demonstrate the reduction in mastoid defect area over time in both adults and children following cochlear implantation. The defect area was measured using high-resolution CT scans of the temporal bone taken at two time points: shortly postoperative (≤ 8 weeks) and > 8 weeks post-surgery. The CI patients were classified into three categories based on the surface area of the mastoid defect: large (exceeding 2 cm^2^), medium (ranging from 1 to 2 cm^2^), and small (less than 1 cm^2^). Initially, adults showed a larger mean mastoid defect area than children. Over time, children exhibited a more pronounced reduction in defect area compared to adults (p < 0.001/p = 0.068), reflecting dynamic changes post-implantation.

At the 1st CT, the difference between children, who were subjected to CT less than 8 weeks after CI versus more than 8 weeks, in terms of small (0% versus 50.4%), medium (29.7% versus 33.9%), and large (70.3% versus 15.7%) defect area was statistically significant (p < 0.001), as shown in Figs. 2 and 3. For adults, the difference between those who were subjected to CT less than 8 weeks after CI versus more than 8 weeks in terms of small (0.0% versus 5.0%), medium (0.0% versus 35.0%), and large (100.0% versus 60.0%) defect area was statistically insignificant (p = 0.083). At the 2nd CT, all children were subjected to the CT more than 8 weeks after the first CT. The percentage of children with small and medium defect areas was 72.2% and 27.8%, respectively. For adults, there were no small defect areas, two had medium defects, and two had large defects, as shown in Table 3.

**Table 3:** Defect areas in relation to the groups and durations.

Groups	The duration between post CI to 1st CT	Total	Defect area						p-value
			Small		Medium		Large		
			N	%	N	%	N	%	
Child	≤ 8 weeks	(N = 37)	0	0.0%	11	29.7%	26	70.3%	**< 0.001**
	> 8 weeks	(N = 115)	58	50.4%	39	33.9%	18	15.7%	
Adult	≤ 8 weeks	(N = 9)	0	0.0%	0	0.0%	9	100.0%	0.083
	> 8 weeks	(N = 20)	1	5.0%	7	35.0%	12	60.0%	
Total	≤ 8 weeks	(N = 46)	0	0%	11	23.91%	35	76.09%	**< 0.001**
	> 8 weeks	(N = 135)	59	43.7%	46	34.07%	30	22.22%	

Group	The duration between 1st CT and 2nd CT	Total	Defect area						p-value
			Small		Medium		Large		
			N	%	N	%	N	%	
Child	≤ 8 weeks	(N = 0)	–	–	–	–	–	–	–
	> 8 weeks	(N = 18)	13	72.2%	5	27.8%	0	0.0%	
Adult	≤ 8 weeks	(N = 2)	0	0.0%	1	50.0%	1	50.0%	0.833
	> 8 weeks	(N = 2)	0	0.0%	1	50.0%	1	50.0%	
Total	≤ 8 weeks	(N = 2)	0	0.0%	1	50.0%	1	50.0%	0.061
	> 8 weeks	(N = 20)	13	65.0%	6	30.0%	1	5.0%	

Bold values indicate statistically significant p-values.

### Relation between the defect area and the study variables

**Figure 4:**
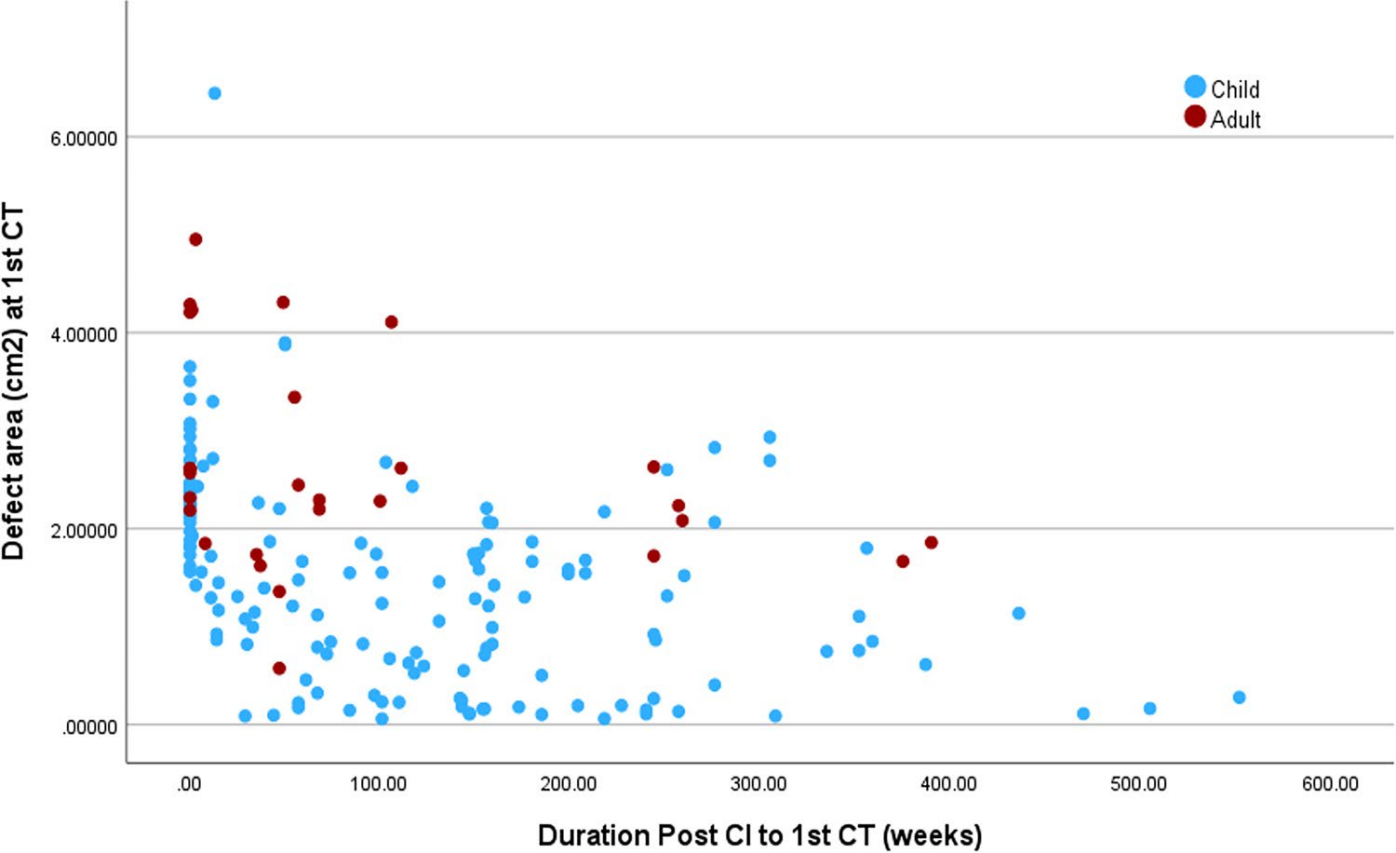
Scatter plot mastoid defect area at the 1st CT against the duration between CI and 1st CT.

The correlation coefficient showed that in the total population, there was a statistically significant negative correlation between defect area at the 1st CT and the duration between post-CI and 1st CT (r = − 0.525; p < 0.001) and age at implantation (r = 0.411; p < 0.001). The correlation between the defect area at the 2nd CT and duration between the 1st and 2nd CT was negative (r = − 0.449; p = 0.036), while the correlation with age at implantation was strong and positive (r = 0.750; p < 0.001). Among the children, there was a significant correlation between defect area at the 1st CT and duration between post-CI and 1st CT (r = − 0.562; p < 0.001) on the one hand, and age at implantation (r = 0.257; p = 0.001), on the other hand. Among the adults, there were no significant correlations between the defect area and the study variables, as shown in Table 4 and Fig. 4.

**Table 4:** Correlation between defect area and the study variables.

Groups	Variables	Defect area at the 1st CT	Defect area at the 2nd CT	Mean difference
All	Duration between post-CI and 1st CT	r = − 0.525; **p < 0.001**	r = − 0.026; p = 0.910	r = 0.504; **p = 0.017**
	Duration between 1st and 2nd CT	r = − 0.299; p = 0.176	r = − 0.449; **p = 0.036**	r = − 0.474; **p = 0.026**
	Age at implantation	r = 0.411; **p < 0.001**	r = 0.750; **p < 0.001**	r = 0.068; p = 0.764
	Side	r = − 0.053; p = 0.477	r = − 0.036; p = 0.872	r = − 0.153; 0.497
Children	Duration between post-CI and 1st CT	r = − 0.562; **p < 0.001**	r = − 0.275; p = 0.270	r = 0.514; **p = 0.029**
	Duration between 1st and 2nd CT	–	–	–
	Age at implantation	r = 0.257; **p = 0.001**	r = 0.554; **p = 0.017**	r = − 0.219; p = 0.383
	Side	r = − 0.090; p = 0.273	r = − 0.172; p = 0.494	r = − 0.172; p = 0.494
Adult	Duration between post-CI and 1st CT	r = − 0.338; p = 0.073	–	–
	Duration between 1st and 2nd CT	–	–	r = − 0.894; p = 0.106
	Age at implantation	r = 0.083; p = 0.668	r = − 0.316; p = 0.684	r = − 0.316; p = 0.684
	Side	r = − 0.059; p = 0.763	r = − 0.775; p = 0.225	r = − 0.258; p = 0.742

Bold values indicate statistically significant p-values.

## Discussion

The present study investigates the differences in mastoid defect area between pediatric and adult populations following CI surgery. Our findings demonstrate the feasibility of measuring mastoid defect area using postoperative TB-CT scans in CI patients. Notably, we observed a significant decrease in mastoid defect area between the initial and follow-up CT scans in children, while no significant change was observed in adults. However, the overall reduction in defect area was comparable in both age groups. Timing of the CT scan emerged as a significant factor, with a higher proportion of larger defect areas found in individuals who underwent the scan within eight weeks after CI. Additionally, a statistically significant correlation was observed between the mastoid defect area and two variables: the age at implantation and the duration between surgery and the first CT scan. The correlation was stronger in children compared to adults.

These findings underscore the importance of considering CT scan timing and age at implantation when assessing mastoid defect areas in CI patients, particularly for revision surgeries. However, it is important to note that there is limited available evidence regarding mastoid bone healing after CI. Our results are consistent with previous studies that emphasize the impact of age on mastoid development, particularly among pediatric individuals^21,22^. Typically, children undergo a gradual growth of their mastoid processes until they reach a size comparable to that observed in adults^1^. Moreover, children tend to demonstrate faster healing, shorter recovery durations, and a lower risk of complications when compared to adults^23^. This can be attributed to factors such as higher cell density, greater regenerative capacity, active bone metabolism, and increased blood flow rates^24–26^. The presence of growth hormone and estrogen at higher levels in children also contributes to their superior bone development and regeneration^27^. It is noteworthy that a prior study emphasizes the continuous mastoid growth observed even in adults^28^.

The accelerated healing of the mastoid bone after CI surgery positively impacts outcomes by reducing the risk of electrode displacement or damage. This study confirmed that even with mastoid regrowth, there was no migration or extrusion of the electrode array^21^. These findings, along with previous research, highlight the importance of placing the electrode lead in a relaxed and coiled configuration within the mastoid cavity. A stable and well-healed mastoid bone provides a solid foundation for accurate placement and effective stimulation of the cochlea, which is vital for a successful CI surgery^29,30^.

During cochlear implantation, the removal of cortical mastoid bone creates a vulnerable area in the mastoid, facilitating the lateral dissemination of infections postoperatively towards the skin rather than the intracranial area. While the greatest susceptibility to infection is observed shortly after surgery, the risk persists beyond the immediate postoperative period, with the majority of cases presenting within the first two years following implantation^16^. A previous study has demonstrated that employing autogenous mastoid cortical bone cap in CI surgery effectively mitigates postoperative complications such as postauricular depressions, and wound infections^9^. However, our findings suggest that this technique may no longer be deemed necessary, particularly in pediatric patients, as the natural regrowth of the mastoid defect on the lateral surface gradually reduces its size over time. On the contrary, in the long-term post-operative phase of CI surgery, careful evaluation is essential when observing post-auricular swelling in patients who have undergone natural mastoid defect closure. It is crucial to consider the possibility of non-surgical mastoiditis or soft tissue swelling as potential causes, instead of solely attributing it to acute otitis media. This is crucial for accurate diagnosis and the implementation of appropriate treatment strategies to address the swelling effectively.

The use of an 8-week cutoff point in the assessment of bone healing is a common practice among clinicians and researchers^31–35^. This is because bone healing is a complex process that can take several weeks to several months, depending on various factors such as the severity of the fracture, the age of the patient, and the presence of underlying medical conditions^36^. In general, it is believed that bone healing is largely complete by eight weeks after a fracture or surgery, although this may vary depending on the individual case. Claes et al. suggested that the remodeling and resorption of periosteal and medullary calluses take between 5 and 8 weeks^4^. The use of an 8-week cutoff point is also practical from a clinical standpoint, as it allows for timely monitoring of bone healing and appropriate treatment decisions to be made.

Despite the valuable insights provided by our study, there are some limitations to acknowledge. Firstly, the timing of post-operative TB-CT was not consistent in all patients, as it is not routinely requested in our center. Additionally, standardizing the CT scan angulation for the oblique sagittal view, which was used to measure the mastoid defect, proved to be challenging due to variations in mastoid process direction based on age and head position.

While the study is an important step in improving our understanding of mastoid bone healing after CI surgery, more research is necessary to confirm the findings and identify potential risk factors for mastoid bone defects post implantation. Moreover, future studies could also investigate the clinical implications of mastoid bone defects post-CI surgery, such as the potential for implant failure or other complications. This information could help clinicians develop more personalized treatment plans for individuals undergoing revision CI surgery and improve the overall outcomes of this procedure.

In conclusion the mastoid defect area can be measured using postoperative CT imaging in patients undergoing cochlear implantation. There are significant differences in mastoid defect regrowth between children and adults after CI surgery. The timing of CT scans and age at implantation were found to be important factors in mastoid defect areas, with a stronger correlation found in children than in adults. These findings contribute to our understanding of mastoid bone development and may have implications for the management of CI patients. Further investigation is warranted to validate these findings, thus facilitating a comprehensive understanding of the factors contributing to mastoid bone regrowth following CI surgery, as well as the long-term changes in mastoid defect area and their clinical significance.

## Data Availability

The datasets used and analyzed during the current study are available from the corresponding author on reasonable request.
